# Mechanical Contribution of the Anterior Talofibular Ligament to Ankle Stability: 3D Anatomical Finite Element Analysis

**DOI:** 10.1002/jor.70241

**Published:** 2026-07-05

**Authors:** Akinobu Minagawa, Naomichi Ogihara, Makoto Kubota, Yuka Matsumoto, Kohta Ito, Tadashi Kimura, Takumi Kihara, Hirotaka Fukushima, Mitsuru Saito

**Affiliations:** ^1^ Department of Orthopaedic Surgery The Jikei University School of Medicine Tokyo Japan; ^2^ Department of Biological Sciences, Graduate School of Science The University of Tokyo Tokyo Japan; ^3^ Research and Development Center Saitama Prefectural University Koshigaya Japan

**Keywords:** ankle instability, anterior talofibular ligament, finite element analysis

## Abstract

Ankle sprains are among the most common musculoskeletal injuries, with the anterior talofibular ligament (ATFL) most frequently affected. Although cadaveric and in vivo studies have demonstrated the ATFL's role in restraining anterior talar translation and inversion, its isolated mechanical contribution remains unclear due to technical challenges in selectively altering individual ligaments. In this study, we developed a three‐dimensional finite element (FE) model of the human foot and ankle to investigate ATFL function. The model was validated against experimental data using the anterior drawer test (ADT), with talar translation in the ATFL‐intact condition falling within reported ranges. Simulations compared ATFL‐intact and ATFL‐injured conditions under ADT, Telos loading, and ankle varus loading. The ATFL‐injured model showed increased anterior talar translation, inversion, and internal rotation, suggesting that the ATFL plays an important role in ankle stability. Fascicular analysis in the present model suggested that the inferior fascicle mainly contributed to resisting anterior translation, whereas the superior fascicle experienced greater model‐predicted loading under inversion and plantar‐flexion positions representative of ankle‐sprain–like postures, suggesting possible vulnerability to injury. These findings provide model‐based biomechanical insight for the ATFL's role in ankle stability and may provide a computational platform for future investigations into chronic ankle instability and surgical reconstruction outcomes.

## 1 Introduction

Ankle sprains are among the most common musculoskeletal injuries in both sports and daily activities, with lateral ligament injuries occurring at particularly high frequency [1, 2]. The lateral ligament complex consists of the anterior talofibular ligament (ATFL), the calcaneofibular ligament (CFL), and the posterior talofibular ligament (PTFL). Within the lateral ligament complex, the ATFL is the most frequently injured structure, either in isolation or in combination with other ligaments [1, 3, 4, 5, 6]. Damage to the ATFL is strongly associated with mechanical instability of the talocrural joint and represents a major contributor to chronic ankle instability (CAI), which increases the risk of recurrent sprains and long‐term degenerative changes [4, 7, 8, 9, 10].

Cadaveric and kinematic studies have demonstrated that the ATFL plays a critical role in restraining anterior translation of the talus and limiting ankle inversion [11, 12, 13]. However, direct in vivo measurement of ATFL loading and its interaction with surrounding ligaments and articular cartilage remains technically challenging, and the detailed distribution of stresses within the joint is still poorly understood [14, 15].

Finite element (FE) analysis offers a powerful means of addressing these limitations by enabling the reconstruction of anatomically accurate three‐dimensional foot and ankle models that incorporate realistic geometry, material properties, and contact interactions. Such models allow estimation of internal stresses, ligament strains, and articular contact mechanics that are otherwise difficult to obtain experimentally. Although FE models of the foot and ankle have been developed to investigate biomechanical function [16, 17, 18, 19, 20], no previous studies have focused on assessing ankle stability at the level of individual ligaments, particularly the ATFL. The mechanical contribution of the ATFL to ankle stability has not been experimentally quantified in isolation, as selectively altering or removing a single ligament in experimental settings is technically challenging without disrupting adjacent structures.

Therefore, the objectives of this study were twofold: (1) to validate an anatomically accurate three‐dimensional FE model of the human foot and ankle by simulating the anterior drawer test (ADT) and comparing the predicted talar displacement with experimental data; and (2) to investigate the mechanical role of the ATFL in ankle stability by comparing model‐predicted ligament forces and joint kinematics during the simulated ADT and the simulated foot inversion occurring due to medial external force between with and without the ATFL conditions. The ADT was chosen for model validation because it is a clinically well‐established and widely used stress test that assesses anterior ankle stability, with talar translation serving as a reliable quantitative indicator of ATFL function [21, 22]. This makes it particularly suitable for testing whether the FE model can reproduce realistic joint kinematics under controlled loading conditions. Applying an external force that induces ankle inversion corresponds to the loading mechanism of ankle sprains, and is therefore suitable for analyzing the contribution of the ATFL to joint stability [23, 24].

## 2 Methods

### 2.1 Ethical Approval

The study protocol was approved by the Institutional Review Board of the Jikei University School of Medicine (approval No. 35‐274(11905)). Written informed consent was obtained from all participants prior to enrollment.

### 2.2 Foot Model

**Figure 1:**
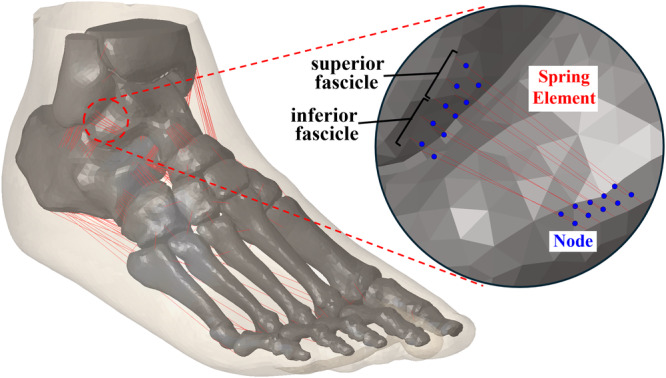
Overview of the entire model and the ATFL model. Bones and articular cartilage are shown in gray. Ligaments and the plantar aponeurosis are shown in red. The ATFL was represented by ten ligament fibers. Each fiber model consisted of four nodes at the origin, insertion, and two intermediate points, with adjacent nodes connected by three spring elements. The five fibers at the proximal attachment were defined as the superior fascicle, and the five distal fibers as the inferior fascicle.

The present study was conducted using an anatomically detailed three‐dimensional FE model of the human foot which was developed and validated in our previous study [25] (Figure 1). The model was reconstructed from CT data of a healthy adult male and consisted of the tibia, fibula, talus, calcaneus, navicular, cuboid, cuneiforms, metatarsals, phalanges, and sesamoids. In addition to the skeletal structures, the model included encapsulated soft tissues, a skin layer, articular cartilage, major foot ligaments, and the plantar aponeurosis, allowing anatomically realistic simulation of internal load transfer within the foot. The bones were represented as isotropic linear elastic materials, whereas the encapsulated soft tissue and skin were modeled using hyperelastic materials. Bones and soft tissues were meshed with tetrahedral elements. Thin cartilaginous layers were added to the articular surfaces and were also meshed with tetrahedral elements. Joint articulation was represented using frictionless surface‐to‐surface contact. The ligaments and the plantar aponeurosis were modeled as tension‐only spring elements. To account for anatomical wrapping and to prevent penetration into bone, ligaments were modeled as series of spring elements interspersed with spherical elements that interacted with the bone surfaces via frictionless contact, allowing sliding along the bone geometry (Figure 1). In addition, major ligaments were represented as bundles of multiple elements to approximate their broad attachment areas. Material properties of the ligaments were defined based on literature values (e.g., Young's modulus ≈ 260 MPa), and their slack lengths were determined relative to the CT‐scanned configuration. Full information regarding image acquisition, mesh construction, material properties, ligament and plantar aponeurosis modeling, boundary conditions, and validation procedures is available in Ito et al. [25].

In this model, the ATFL was divided into superior and inferior fascicles, consistent with previous anatomical descriptions [23, 26, 27]. The intact foot model was defined as the ATFL‐intact model, while the model in which ATFL tensile force was set to zero was defined as the ATFL‐injured model. In this study, continuum representations of ligaments were not used because the present aim was to assess the macroscopic stabilizing role of the ATFL. This function can be adequately represented using spring‐based elements, which also allow explicit definition of slack length, whereas continuum models require additional assumptions that can affect ligament recruitment.

All explicit dynamic simulations were conducted on a high‐performance computing system (Wisteria/BDEC‐01 “Odyssey”; Information Technology Center, The University of Tokyo, Tokyo, Japan) using an explicit finite element solver (RADIOSS, HyperWorks 2022.3.0; Altair Engineering, Troy, MI, USA).

### 2.3 ADT Validation

**Figure 2:**
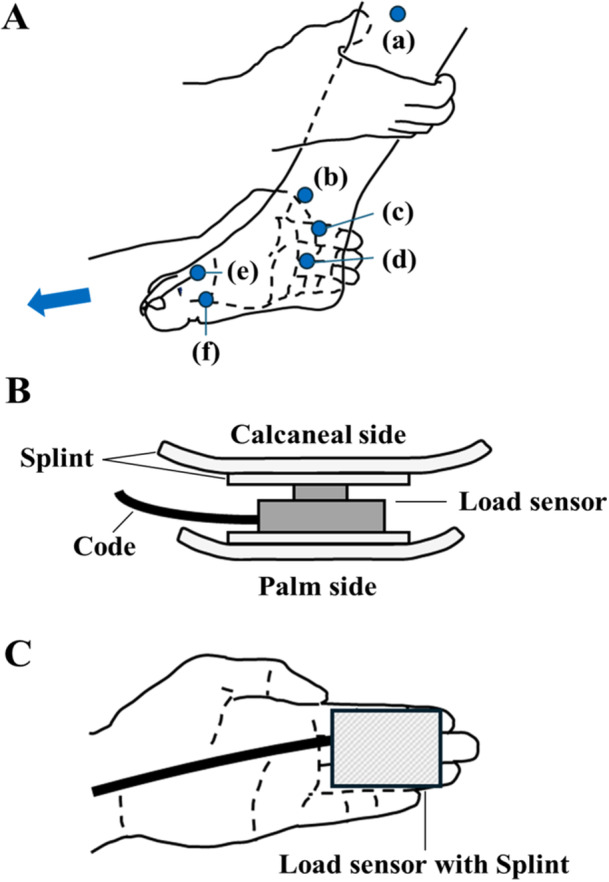
Experimental setup of the ADT. (A) Marker placement for motion capture during the ADT. (a) Right tibial tuberosity, (b) Lateral malleolus, (c) Medial malleolus, (d) Sustentaculum tali, (e) Head of the second metatarsal, (f) Medial aspect of the head of the first metatarsal. (B) Structure of the load measurement system. The load sensor was secured between two splints of different sizes to ensure uniform force transmission. (C) Positioning of the load sensor. The sensor was placed distal to the second through fourth metacarpophalangeal (MP) joints of the left hand.

For reproducing the ADT and comparing it with experimental data, we first measured the ADT performed by a board‐certified orthopedic surgeon (7 years of clinical experience) on two healthy adult participants without a history of ankle sprain, using a motion capture system (MAC3D; Motion Analysis Corporation, Santa Rosa, CA, USA) and a digital force gauge (eCLFX‐500N with eZT, IMADA, Toyohashi, Japan). In the ADT, the examiner stabilized the distal tibia with one hand and pulled the calcaneus forward with the other to assess anterior translation of the talus. Foot motion was quantified by placing reflective markers at six anatomical landmarks: (a) tibial tuberosity, (b) lateral malleolus, (c) medial malleolus, (d) sustentaculum tali, (e) head of the second metatarsal, and (f) medial aspect of the head of the first metatarsal (Figure 2A). Marker movements were recorded at 100 Hz. Simultaneously, the external force applied to the calcaneal tuberosity during the ADT was recorded at 100 Hz. To ensure accurate measurement of the force transmitted from the examiner's hand to the foot, the sensor was secured between soft splints and positioned on the examiner's palm (Figure 2B,C).

**Figure 3:**
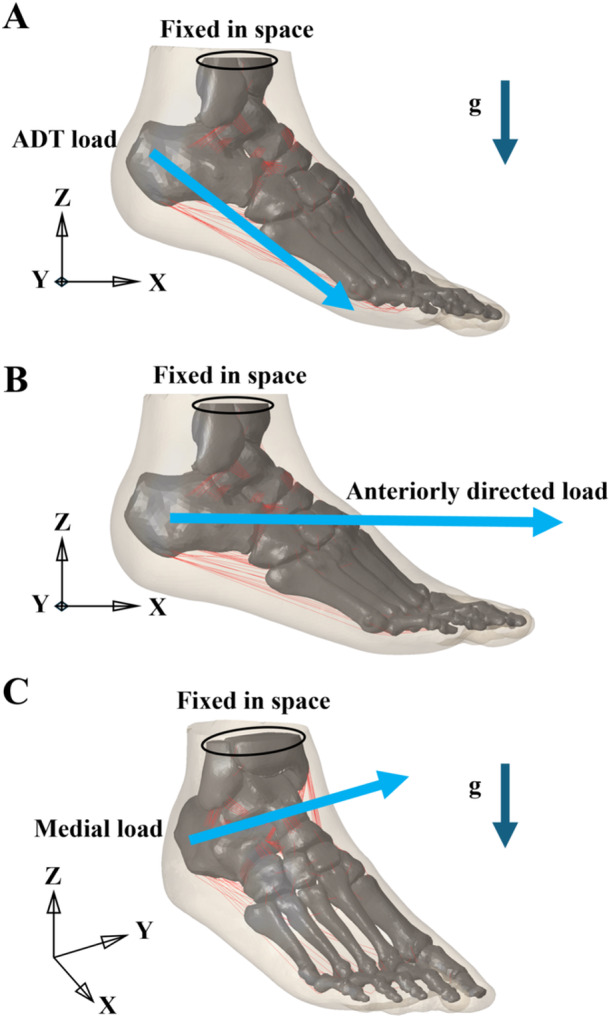
Boundary conditions applied in each simulation. (A) ADT simulation. The lower leg was fixed in space, and gravitational acceleration of 9.81 m/s^2^ was applied to the entire model. In addition, an ADT load was applied to the calcaneus in a direction parallel to the long axis of the second metatarsal. (B) Telos simulation. The lower leg was fixed, and calcaneal plantarflexion and dorsiflexion were constrained. An anteriorly directed load was applied to the calcaneus along the global X‐axis. (C) Foot inversion simulation. The lower leg was fixed, gravitational acceleration of 9.81 m/s^2^ was applied to the entire model, and a medial load was applied to the calcaneus along the global Y‐axis.

In the ADT simulation, the tibia and fibula were fixed in the space and natural plantarflexion of the foot due to the gravitational force was calculated. This was achieved by constraining translational motion and rotation at the nodes on the superior surfaces of the tibia and fibula and by applying gravitational acceleration (9.81 m/s^2^) to the entire model. From this posture, a time‐varying external force based on the measured force profile was applied to all calcaneal nodes in a direction parallel to the long axis of the second metatarsal (Figure 3A). The load profile was modeled by fitting a Gaussian curve to the experimental waveform and applied accordingly.

Anterior talar displacement is typically defined as the distance between the posterior edge of the tibial plafond and the nearest point on the articular cartilage of the talar trochlea [22, 28]. However, for the comparisons with the ADT experimental data, anterior movement of the talus during the ADT was quantified as the increase in distance between the sustentaculum tali and the midpoint of the medial and lateral malleoli from the onset of force application. Because the talus lacks palpable external landmarks [29], the sustentaculum tali, which moves synchronously with the talus through the subtalar joint, was used as a surrogate marker of talar motion. This surrogate measure was used solely for validation against the experimental ADT data.

### 2.4 Telos Stress Validation

To further validate the FE model, the loading conditions of a previous Telos stress radiography study [30] were reproduced and anterior talar translation under a 150 N load was compared between experimentally obtained and simulated values both in the ATFL‐intact and ATFL‐injured conditions. Because no reference material describing the detailed loading profile of the Telos device could be identified, the load was applied as a simplified function that reached 150 N at 10 ms after the onset of loading and was then maintained thereafter. As in the ADT simulation, the tibia and fibula were fixed in space, and a total anteriorly directed force of 150 N was applied to the calcaneus by distributing it over all calcaneal nodes (Figure 3B). In the Telos device, the heel is fixed in a foot‐holding apparatus. Therefore, in the simulation, rotation of the calcaneus about the global Y‐axis, corresponding to the plantarflexion–dorsiflexion axis, was constrained. Because no obvious motion was observed after 500 ms from the start of the analysis, the results at 500 ms were used for comparison. Anterior talar translation was quantified by measuring the shortest sagittal projection distance between the posterior tibial plafond and the closest point on the talar articular cartilage. Both ATFL‐intact and ATFL‐injured models were simulated, and the difference in translation between models was defined as the difference in anterior translation, consistent with the literature [30].

### 2.5 Comparisons Between ATFL‐Intact and ATFL‐Injured Models

This study investigated the mechanical role of the ATFL in ankle stability by comparing joint kinematics and model‐predicted ligament forces during (1) a simulated ADT induced by an anterior external force, (2) a simulated Telos test, and (3) a simulated foot inversion induced by a medial external force, under conditions with and without the ATFL. For the ADT and Telos test, the same simulation protocol used for validation (described above) was adopted under the ATFL‐injured condition and the resulting joint kinematics and ligament forces were compared between the intact and ATFL‐deficient conditions. For the foot inversion, a medial external force was applied to induce inversion of the foot and the resulting joint kinematics and ligament forces were compared between the two conditions. Specifically, the tibia and fibula were fixed in space, and the natural plantarflexed posture of the foot was established under gravity. A total medially directed force of 100 N was applied to the calcaneus by distributing it over all calcaneal nodes (Figure 3C), based on a previous study [31]. The load was applied as a simplified step function so that 100 N was applied from the onset of loading. Because no obvious motion was observed after 300 ms from the start of the analysis, the results at 300 ms were used for comparison. A timescale of this order is not considered unreasonable for a short‐duration ankle loading condition in ankle sprain [32].

### 2.6 Talocrural Joint Kinematics

In order to characterize the rotational movement of the talus with respect to the tibia in all simulations, a bone fixed local coordinate system was defined as in Figure 3, such that the three orthonormal axes (x, y, z) of the local coordinate system at the onset of force application were aligned with the three orthonormal axes (X, Y, Z) of the global coordinate system [33]. The X‐, Y‐, and Z‐axes roughly corresponded to the inversion–eversion, plantarflexion–dorsiflexion, and internal–external rotation axes, respectively. The joint angles were computed as Euler angles using the y–x–z order.

### 2.7 Ligament Forces

Ligament force has been widely used as an indicator to evaluate the mechanical contribution of ligaments to ankle joint stability [34, 35]. In the present study, therefore, model‐based estimates of forces in the superior and inferior fascicles of the ATFL, as well as in the CFL, and the PTFL, were evaluated to assess the mechanical contributions of the lateral ligaments and the potential redistribution of ligament loading following ATFL injury. In the ATFL‐intact model, forces in all ligaments were calculated. In the ATFL‐injured model, the ATFL force was set to zero; therefore, the forces in the CFL and PTFL were compared between the ATFL‐intact and ATFL‐injured models. Ligament and fascicle forces were calculated as the sums of the forces of their constituent fibers.

## 3 Results

**Figure 4:**
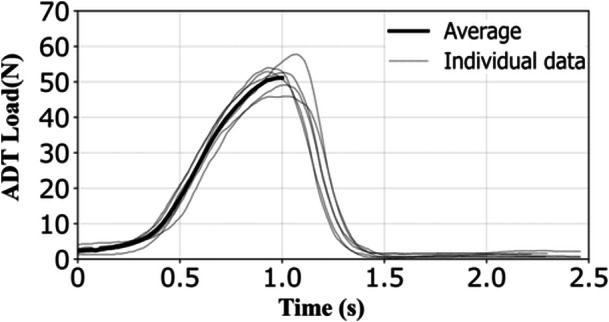
ADT loading waveform from experiments. Load waveform obtained from ADT experiments. Thin lines show waveforms from individual trials and the thick line indicates the average waveform of the trials. The time to peak was approximately 1.0 s, and the mean peak force was 52.1 N. The average waveform was fitted with a Gaussian function, and the fitted curve was used as the input load for the simulation.

The force profiles applied to the calcaneal tuberosity during the ADT, aligned at the onset of force application, were consistent across the two participants and among the three trials, exhibiting a Gaussian‐like curve (Figure 4). Because the present validation aimed to assess whether the model reproduced a representative mechanical response under the ADT, rather than variability across trials and subjects, an average waveform was computed and fitted with a Gaussian function, which was subsequently applied to the finite element model. The peak load of the averaged waveform was 52.1 N. The mean surrogate anterior talar translation during the ADT across six trials was 4.39 ± 0.88 mm, while the corresponding surrogate value obtained from the simulation was 4.9 mm.

In the simulated Telos stress test, the anterior talar translations were 7.2 mm and 8.9 mm for the ATFL‐intact and ATFL‐injured models, respectively. The corresponding values reported in the published experiment [30] were 5.7 ± 2.3 mm and 7.4 ± 2.4 mm, respectively. Thus, the difference in anterior talar translation between the ATFL‐intact and ATFL‐injured models was 1.7 mm in the simulation and 1.7 ± 1.9 mm in the published experiment.

**Figure 5:**
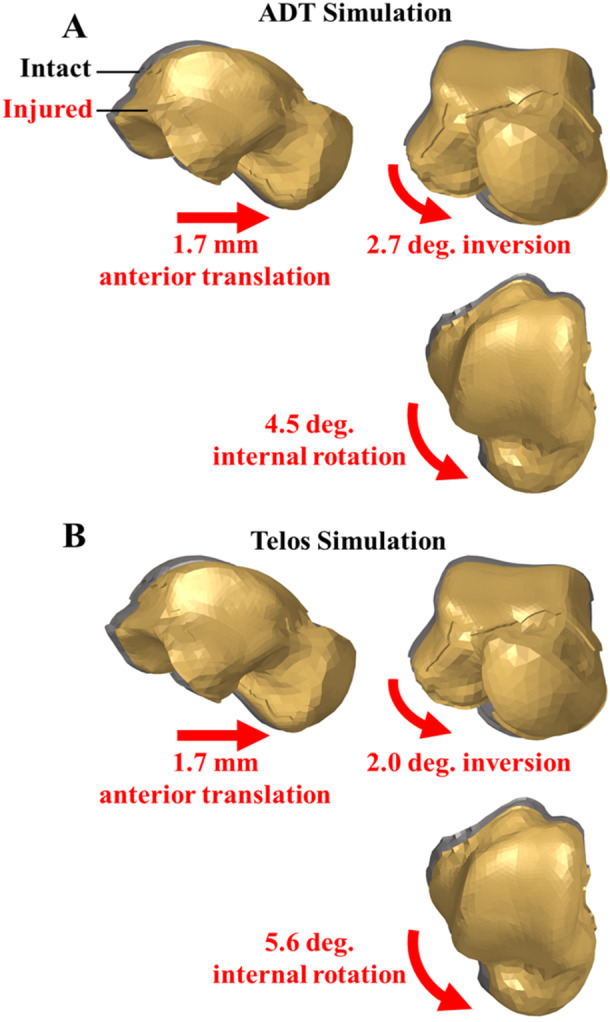
Comparison of talar posture and angles in the ADT and Telos simulations. Talar posture and changes in angles relative to the tibia were compared between ATFL‐intact (gray) and ATFL‐injured (yellow ochre) models. (A) ADT simulation. In the injured model, the talus translated 1.7 mm more anteriorly, inversion increased by 2.7°, and internal rotation increased by 4.5° compared with the intact model. (B) Telos simulation. In the injured model, the talus translated 1.7 mm more anteriorly, inversion increased by 2.0°, and internal rotation increased by 5.6° compared with the intact model.

When comparing the ATFL‐intact and ATFL‐injured models under ADT loading, differences in foot motion were observed despite the application of the same external force (Figure 5A). The anterior talar translation was 1.1 mm in the ATFL‐intact model and 2.8 mm in the ATFL‐injured model, resulting in a difference of 1.7 mm between the two models. In the ATFL‐intact model, the talocrural joint was dorsiflexed by 7.4°, inverted by 0.8°, and externally rotated by 2.0°, whereas the corresponding angles for the ATFL‐injured model were 7.4°, 3.5°, and −2.5°, respectively (negative indicating internal rotation). Additionally, in the present simulation, relative to the intact model, the ATFL‐injured model showed larger tibiotalar inversion and internal rotation by 2.7° and 4.5°, respectively. In the intact model, model‐predicted ligament tension was 0 N in the superior ATFL fascicle, 47 N in the inferior fascicle, 7 N in the CFL, and 54 N in the PTFL. In the injured model, model‐predicted ligament tension increased to 16 N in the CFL but decreased to 35 N in the PTFL. These results should be interpreted as within‐model comparisons, suggesting a possible change in ligament load sharing after ATFL injury.

In the Telos test, in the ATFL‐intact model, the talocrural joint was dorsiflexed by 1.9°, inverted by −0.4° (negative indicating eversion), and externally rotated by 2.0°, whereas the corresponding angles for the ATFL‐injured model were 1.8°, 1.6°, and −3.6°, respectively (negative indicating internal rotation) (Figure 5B). In the present simulation, tibiotalar inversion was 2.0° greater and internal rotation was 5.6° greater in the injured model than in the intact model. Regarding model‐predicted ligament tension, the intact model showed 14 N in the superior ATFL fascicle, 95 N in the inferior fascicle, 0 N in the CFL, and 39 N in the PTFL. In the injured model, CFL tension was 0 N (unchanged) and PTFL tension increased to 139 N.

**Figure 6:**
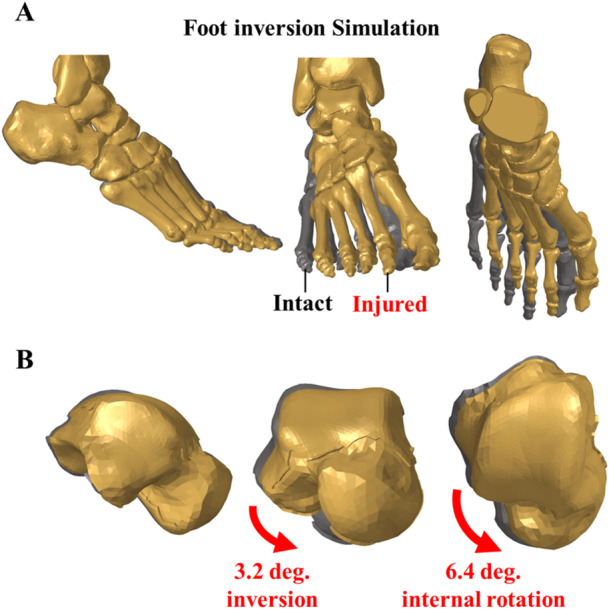
Comparison of foot and talar posture and angles in the foot inversion simulation. Foot and talar posture relative to the tibia were compared between ATFL‐intact (gray) and ATFL‐injured (yellow ochre) models. (A) The injured model showed greater inversion and internal rotation of the foot compared with the intact model. (B) At the talocrural joint, inversion increased by 3.2° and internal rotation increased by 6.4° in the injured model compared with the intact model.

Under the foot inversion simulation, the injured model exhibited larger inversion and internal rotation of the entire foot than the intact model (Figure 6A). At the tibiotalar joint, inversion increased by 3.2° and internal rotation by 6.4° relative to the intact model (Figure 6B). For model‐predicted ligament tension, the intact model showed 67 N and 14 N in the superior and inferior ATFL fascicles, 67 N in the CFL, and 78 N in the PTFL, whereas the injured model showed 80 N in the CFL and 84 N in the PTFL.

## 4 Discussion

Our FE model reproduced both ATFL‐intact and ATFL‐injured conditions and was validated primarily by comparing anterior talar translation with experimental data. The model also showed kinematic trends that were broadly consistent with those reported in previous studies. In the ADT simulation, talar displacement in the ATFL‐intact model fell within the range reported in vivo, and in the Telos simulation both the magnitude of anterior translation and the difference between intact and injured conditions were close to previously published values [30]. These findings suggest that the present model can reasonably reproduce anterior talar translation under clinically relevant loading conditions. Because only the ATFL condition was altered while all other factors were held constant, the observed differences can be interpreted as the isolated mechanical contribution of the ATFL within this model.

In the present simulation, the injured model exhibited greater anterior talar translation, inversion, and internal rotation than the intact model under both ADT and Telos loading. These results are consistent with in vivo studies of patients with lateral ankle instability demonstrating excessive anterior translation and internal rotation of the talus [11], and with cadaveric robotic studies showing that sectioning the ATFL reduces resistance to anterior translation, inversion, and internal rotation [12]. Moreover, model‐predicted ligament force analysis suggested that the ATFL sustained greater tension than the other lateral ligaments across several loading conditions, indicating an important mechanical role in stabilizing the talocrural joint, particularly in resisting anterior translation and medial loading. Clinically, these findings may underlie the tendency for recurrent ankle sprains and the development of CAI. However, because PTFL tension was similar to that of the ATFL in some conditions and the present study did not compare stability outcomes under other lateral ligament injury conditions or progressive lateral ligament injury conditions, whether the ATFL is the primary stabilizer of the ankle should be interpreted with care. Therefore, a systematic comparison of ankle stability under such conditions will be an important subject for future work.

Fascicular analysis of the ATFL provided further insight. In the foot inversion simulation, which reproduced the posture of a lateral ankle sprain, the superior fascicle experienced greater tension than the inferior fascicle. Because isolated injury of the superior fascicle can produce instability [12, 36], and because anatomical studies show it to be longer, wider, and thicker than the inferior fascicle [26, 27, 37, 38], this fascicle likely plays a major role in ankle stability. By contrast, in both the ADT and Telos simulations, the inferior fascicle carried greater tension. These clinical maneuvers are widely used to evaluate ankle instability and to guide ATFL reconstruction for CAI, but our findings suggest that they mainly load the inferior fascicle and may therefore underestimate instability when the superior fascicle alone is injured. This is consistent with the concept of micro‐instability and supports reports that patients may show instability symptoms despite negative ADT findings [9, 32, 39]. A more accurate assessment of instability will require test positions and external loads that approximate those of an actual sprain. The anterolateral drawer and reverse anterolateral drawer tests have been proposed [40, 41], but like the conventional ADT, both involve less plantarflexion than the typical sprain posture and may not adequately evaluate the superior fascicle.

In addition, the foot inversion simulation demonstrated that the CFL and PTFL exhibited increased tensile forces in the ATFL‐injured model compared with the intact model. This finding may explain a clinical observation that patients with CAI sustaining recurrent sprains often report pain in the region of the PTFL. The present results suggest that once the ATFL is weakened, compensatory loading is shifted onto the CFL and PTFL during inversion, with increased model‐predicted loading on these ligaments. Although direct clinical or experimental evidence on this phenomenon has not been reported to our knowledge, the present simulation indicates a potential mechanism for recurrent symptoms in CAI patients. These findings suggest a possible redistribution of mechanical loads among the lateral ankle ligaments after ATFL injury and may help to better understand pain localization in CAI and to guide preventive and rehabilitative strategies.

Finally, our model enables systematic exploration across postures, load directions, and concomitant CFL injury. It also suggest that the ATFL should not be regarded as a single, independent ligament, but rather as two fascicles that together form an integral part of the lateral ligament complex. Considering fascicular structure will therefore be important to restore physiological load sharing and stability in CAI.

This study has several limitations. First, the FE model was created from CT data of one healthy adult and was not participant‐specific; no subject‐specific geometric scaling was performed, and validation was conducted at the generalized‐model level using data from two participants, which limits generalizability. Second, ADT measurements used for validation were obtained by a single examiner, and experimental talar translation was quantified using the surrogate measure, potentially introducing measurement error. Third, ligaments were modeled as simplified spring elements as in previous studies [42, 43, 44] rather than continuum representations, and ligament tension was not directly validated against experimental ligament tension data. Although the model was validated against anterior talar translation under ADT and Telos loading conditions, agreement in global kinematics does not necessarily ensure the accuracy of ligament force predictions. Therefore, detailed stress–strain behavior and absolute force magnitudes should be interpreted with caution. In addition, a systematic sensitivity analysis and parameter tuning (e.g., ligament stiffness) were not performed, and thus the influence of modeling assumptions on the predicted joint kinematics cannot be excluded. Finally, because only the ATFL‐deficient condition was examined, the relative contributions of other lateral ligaments were not systematically evaluated. Further studies incorporating progressive ligament injury conditions are needed.

## 5 Conclusion

We developed an anatomically detailed three‐dimensional FE model of the human foot and ankle and used it to examine the role of the ATFL in ankle stability. The model validated primarily with respect to anterior talar translation under both ATFL‐intact and ATFL‐injured conditions and provided model‐based estimates indicating that the ATFL makes an important contribution to resisting anterior translation, inversion, and internal rotation of the talus. Loss of the ATFL was associated with increased ankle instability in the present simulations. These results provide insight into the possible mechanical role of the ATFL in ankle stability and may be useful for understanding the functional consequences of ATFL injury and for improving approaches to ligament repair and reconstruction.

## Author Contributions

Akinobu Minagawa, Naomichi Ogihara, and Makoto Kubota conceived and designed the study. Akinobu Minagawa performed the simulations and data analysis. Naomichi Ogihara, Makoto Kubota, Tadashi Kimura, and Hirotaka Fukushima evaluated the simulation results. Yuka Matsumoto and Kohta Ito refined the finite element model. Akinobu Minagawa and Takumi Kihara conducted the experimental measurements. Naomichi Ogihara, Makoto Kubota and Mitsuru Saito supervised the overall study. Akinobu Minagawa drafted the manuscript. Naomichi Ogihara, Makoto Kubota, and Mitsuru Saito critically revised the manuscript. All authors read and approved the final version of the manuscript.

## Conflicts of Interest

The authors declare no conflicts of interest.

## Data Availability

The data that support the findings of this study are available on request from the corresponding author. The data are not publicly available due to privacy or ethical restrictions.

## References

[1] D. T. P. Fong , Y. Hong , L. K. Chan , P. S. H. Yung , and K. M. Chan , “A Systematic Review on Ankle Injury and Ankle Sprain in Sports,” Sports Medicine 37 (2007): 73–94. 17190537 10.2165/00007256-200737010-00006

[2] C. W. DiGiovanni and A. Brodsky , “Current Concepts: Lateral Ankle Instability,” Foot & Ankle International 27 (2006): 854–866. 17054892 10.1177/107110070602701019

[3] P. A. Renström and L. Konradsen , “Ankle Ligament Injuries,” British Journal of Sports Medicine 31 (1997): 11–20. 9132202 10.1136/bjsm.31.1.11 PMC1332467

[4] M. M. Herzog , Z. Y. Kerr , S. W. Marshall , and E. A. Wikstrom , “Epidemiology of Ankle Sprains and Chronic Ankle Instability,” Journal of Athletic Training 54 (2019): 603–610. 31135209 10.4085/1062-6050-447-17 PMC6602402

[5] P. D'Hooghe , F. Cruz , and K. Alkhelaifi , “Return to Play After a Lateral Ligament Ankle Sprain,” Current Reviews in Musculoskeletal Medicine 13 (2020): 281–288. 32377961 10.1007/s12178-020-09631-1 PMC7251008

[6] J. S. Kim , M. S. Kim , D. K. Kim , and S. H. Lee , “Magnetic Resonance Imaging Characteristics of a Lateral Ligament Injury in Acute Ankle Sprains Among Athletes,” Orthopaedic Journal of Sports Medicine 11 (2023): 23259671231207688. 37954866 10.1177/23259671231207688 PMC10637175

[7] A. Alajlan , S. Santini , F. Alsayel , et al., “Joint‐Preserving Surgery in Varus Ankle Osteoarthritis,” Journal of Clinical Medicine 11 (2022): 2194. 35456287 10.3390/jcm11082194 PMC9031025

[8] R. Krips , J. de Vries , and C. N. van Dijk , “Ankle Instability,” Foot and Ankle Clinics 11 (2006): 311–329. 16798514 10.1016/j.fcl.2006.02.003

[9] C. C. Hong , K. J. Tan , and J. Calder , “Chronic Lateral Ankle Ligament Instability—Current Evidence and Recent Management Advances,” Journal of Clinical Orthopaedics and Trauma 48 (2024): 102328. 38274643 10.1016/j.jcot.2023.102328 PMC10806209

[10] P. A. Gribble , C. M. Bleakley , B. M. Caulfield , et al., “Evidence Review for the 2016 International Ankle Consortium Consensus Statement on the Prevalence, Impact and Long‐Term Consequences of Lateral Ankle Sprains,” British Journal of Sports Medicine 50 (2016): 1496–1505. 27259753 10.1136/bjsports-2016-096189

[11] A. M. Caputo , J. Y. Lee , C. E. Spritzer , et al., “In Vivo Kinematics of the Tibiotalar Joint After Lateral Ankle Instability,” American Journal of Sports Medicine 37 (2009): 2241–2248. 19622791 10.1177/0363546509337578 PMC2891039

[12] M. Dalmau‐Pastor , H. El‐Daou , J. M. Stephen , J. Vega , F. Malagelada , and J. Calder , “Clinical Relevance and Function of Anterior Talofibular Ligament Superior and Inferior Fascicles: A Robotic Study,” American Journal of Sports Medicine 51 (2023): 2169–2175. 37232327 10.1177/03635465231172196

[13] Y. Ruan , S. Wang , N. Zhang , et al., “In Vivo Analysis of Ankle Joint Kinematics and Ligament Deformation of Chronic Ankle Instability Patients During Level Walking,” Frontiers in Bioengineering and Biotechnology 12 (2024): 1441005. 39165404 10.3389/fbioe.2024.1441005 PMC11333339

[14] Q. Zhang , N. C. Adam , S. H. Hosseini Nasab , W. R. Taylor , and C. R. Smith , “Techniques for In Vivo Measurement of Ligament and Tendon Strain: A Review,” Annals of Biomedical Engineering 49 (2021): 7–28. 33025317 10.1007/s10439-020-02635-5 PMC7773624

[15] J. E. Bischof , C. E. Spritzer , A. M. Caputo , et al., “In Vivo Cartilage Contact Strains in Patients With Lateral Ankle Instability,” Journal of Biomechanics 43 (2010): 2561–2566. 20605154 10.1016/j.jbiomech.2010.05.013 PMC3031910

[16] L. Peng , L. Yu , J. Jia , et al., “The Effect of Thickness and Elastic Modulus of the Anterior Talofibular Ligament on Anterior Ankle Joint Stiffness: A Subject‐Specific Finite Element Study,” Frontiers in Bioengineering and Biotechnology 11 (2023): 1175347. 37180042 10.3389/fbioe.2023.1175347 PMC10166853

[17] Z. Zhou , H. Zhou , T. Jie , et al., “Analysis of Stress Response Distribution in Patients With Lateral Ankle Ligament Injuries: A Study of Neural Control Strategies Utilizing Predictive Computing Models,” Frontiers in Physiology 15 (2024): 1438194. 39113939 10.3389/fphys.2024.1438194 PMC11303170

[18] C. W. Wang , A. Muheremu , and J. P. Bai , “Use of Three‐Dimensional Finite Element Models of the Lateral Ankle Ligaments to Evaluate Three Surgical Techniques,” Journal of International Medical Research 46 (2018): 699–709. 29239256 10.1177/0300060517727941 PMC5971510

[19] L. Zhang , R. Wang , S. Yang , et al., “Anterior Talofibular Ligament Repair in Combination With Anterior Tibiofibular Ligament Distal Fascicle Transfer for The Treatment of Chronic Lateral Ankle Instability: A Finite Element Analysis,” Journal of Foot and Ankle Surgery 63 (2024): 435–442. 10.1053/j.jfas.2024.02.001 38438102

[20] N. Mercan , A. Yurteri , and Y. Dere , “Do Lateral Ankle Ligaments Contribute to Syndesmotic Stability: A Finite Element Analysis Study,” Computer Methods in Biomechanics and Biomedical Engineering 27 (2024): 1768–1780. 37728074 10.1080/10255842.2023.2258251

[21] F. Netterström‐Wedin , M. Matthews , and C. Bleakley , “Diagnostic Accuracy of Clinical Tests Assessing Ligamentous Injury of the Talocrural and Subtalar Joints: A Systematic Review With Meta‐Analysis,” Sports Health: A Multidisciplinary Approach 14 (2022): 336–347. 10.1177/19417381211029953 PMC9109591 34286639

[22] K. Kamada , Y. Hoshino , T. Yamamoto , M. Kamachi , N. Kanzaki , and R. Kuroda , “Diagnostic Strategies for Chronic Lateral Ankle Instability: A Narrative Review,” Annals of Joint 9 (2024): 41. 39540064 10.21037/aoj-24-31 PMC11558280

[23] P. Golanó , J. Vega , P. A. J. de Leeuw , et al., “Anatomy of the Ankle Ligaments: A Pictorial Essay,” Knee Surgery, Sports Traumatology, Arthroscopy 18 (2010): 557–569. 10.1007/s00167-010-1100-x PMC2855022 20309522

[24] E. S. Hur , D. D. Bohl , and S. Lee , “Lateral Ligament Instability: Review of Pathology and Diagnosis,” Current Reviews in Musculoskeletal Medicine 13 (2020): 494–500. 32495041 10.1007/s12178-020-09641-z PMC7340720

[25] K. Ito , Y. Matsumoto , H. Seki , T. Nagura , and N. Ogihara , “Simulating Human Foot Mechanics During Walking Based on an Anatomically Detailed Forward Dynamic Finite Element Model,” Annals of Biomedical Engineering 54 (2026): 1435–1449. 41530651 10.1007/s10439-026-03984-3 PMC13091900

[26] H. Yang , M. Su , Z. Chen , et al., “Anatomic Measurement and Variability Analysis of the Anterior Talofibular Ligament and Calcaneofibular Ligament of the Ankle,” Orthopaedic Journal of Sports Medicine 9 (2021): 23259671211047269. 34820459 10.1177/23259671211047269 PMC8607490

[27] M. Edama , I. Kageyama , T. Kikumoto , et al., “Morphological Features of the Anterior Talofibular Ligament by the Number of Fiber Bundles,” Annals of Anatomy ‐ Anatomischer Anzeiger 216 (2018): 69–74. 29196235 10.1016/j.aanat.2017.11.001

[28] B. D. Beynnon , G. Webb , B. M. Huber , C. N. Pappas , P. Renström , and L. D. Haugh , “Radiographic Measurement of Anterior Talar Translation in the Ankle: Determination of the Most Reliable Method,” Clinical Biomechanics 20 (2005): 301–306. 15698703 10.1016/j.clinbiomech.2004.11.011

[29] M. A. Hegazy , H. M. Khairy , A. A. Hegazy , M. A. E. F. Sebaei , and S. I. Sadek , “Talus Bone: Normal Anatomy, Anatomical Variations and Clinical Correlations,” Anatomical Science International 98 (2023): 391–406. 37017903 10.1007/s12565-023-00712-y

[30] J. H. Cho , D. H. Lee , H. K. Song , J. Y. Bang , K. T. Lee , and Y. U. Park , “Value of Stress Ultrasound for the Diagnosis of Chronic Ankle Instability Compared to Manual Anterior Drawer Test, Stress Radiography, Magnetic Resonance Imaging, and Arthroscopy,” Knee Surgery, Sports Traumatology, Arthroscopy 24 (2016): 1022–1028. 10.1007/s00167-015-3828-9 26515772

[31] L. H. Gimber , L. D. Latt , C. Caruso , et al., “Ultrasound Shear Wave Elastography of the Anterior Talofibular and Calcaneofibular Ligaments in Healthy Subjects,” Journal of Ultrasonography 21 (2021): e86–e94. 34258033 10.15557/JoU.2021.0017 PMC8264467

[32] E. Kristianslund , R. Bahr , and T. Krosshaug , “Kinematics and Kinetics of an Accidental Lateral Ankle Sprain,” Journal of Biomechanics 44 (2011): 2576–2578. 21824618 10.1016/j.jbiomech.2011.07.014

[33] K. Ito , T. Nakamura , R. Suzuki , et al., “Comparative Functional Morphology of Human and Chimpanzee Feet Based on Three‐Dimensional Finite Element Analysis,” Frontiers in Bioengineering and Biotechnology 9 (2021): 760486. 35096789 10.3389/fbioe.2021.760486 PMC8793834

[34] K. Takahashi , A. Teramoto , Y. Murahashi , et al., “The In Situ Force and Contribution of Each Ligamentous Band of the Deltoid Ligament in Ankle Joint Stability: A Cadaveric Biomechanical Study,” Orthopaedic Journal of Sports Medicine 13 (2025): 23259671251327406. 40182569 10.1177/23259671251327406 PMC11963784

[35] T. Kobayashi , S. Yamakawa , K. Watanabe , et al., “The In Situ Force in the Calcaneofibular Ligament and the Contribution of This Ligament to Ankle Joint Stability,” Clinical Biomechanics 40 (2016): 8–13. 27771606 10.1016/j.clinbiomech.2016.10.009

[36] J. Vega , F. Malagelada , and M. Dalmau‐Pastor , “Ankle Microinstability: Arthroscopic Findings Reveal Four Types of Lesion to the Anterior Talofibular Ligament's Superior Fascicle,” Knee Surgery, Sports Traumatology, Arthroscopy 29 (2021): 1294–1303. 10.1007/s00167-020-06089-z 32518964

[37] A. Kakegawa , N. Fukushima , N. Sumitomo , A. Nagira , and Y. Ichinose , “Difference in the Fibular Attachment Structure between the Superior and Inferior Fascicles of the Anterior Talofibular Ligament Using Ultrasonography and Histological Examinations,” Surgical and Radiologic Anatomy 44 (2022): 1513–1520. 36449085 10.1007/s00276-022-03049-9

[38] Z. Chen , H. Yang , X. Zhang , et al., “Anatomical Analysis of the Lateral Ligament Complex in the Neutral Position and During Plantar Flexion,” BMC Musculoskeletal Disorders 26 (2025): 445. 40329254 10.1186/s12891-025-08699-5 PMC12054227

[39] J. Vega and M. Dalmau‐Pastor , “Ankle Joint Microinstability,” Foot and Ankle Clinics 28 (2023): 333–344. 37137627 10.1016/j.fcl.2023.01.008

[40] P. Phisitkul , C. Chaichankul , R. Sripongsai , I. Prasitdamrong , P. Tengtrakulcharoen , and S. Suarchawaratana , “Accuracy of Anterolateral Drawer Test in Lateral Ankle Instability: A Cadaveric Study,” Foot & Ankle International 30 (2009): 690–695. 19589318 10.3113/FAI.2009.0690

[41] Q. Li , Y. Tu , J. Chen , et al., “Reverse Anterolateral Drawer Test Is More Sensitive and Accurate for Diagnosing Chronic Anterior Talofibular Ligament Injury,” Knee Surgery, Sports Traumatology, Arthroscopy 28 (2020): 55–62. 10.1007/s00167-019-05705-x 31559464

[42] S. Ji , L. Sun , Q. Wang , et al., “The Impact of Lateral Ankle Ligament Injuries on Ankle Stability and Talar Cartilage Stress: A Finite Element Analysis of Combined Injury Mechanisms,” Frontiers in Bioengineering and Biotechnology 13 (2025): 1697096. 41194982 10.3389/fbioe.2025.1697096 PMC12583215

[43] H. Malakoutikhah , E. Madenci , and L. D. Latt , “The Impact of Ligament Tears on Joint Contact Mechanics in Progressive Collapsing Foot Deformity: A Finite Element Study,” Clinical Biomechanics 94 (2022): 105630. 35334403 10.1016/j.clinbiomech.2022.105630

[44] H. Malakoutikhah , E. Madenci , and L. D. Latt , “The Contribution of the Ligaments in Progressive Collapsing Foot Deformity: A Comprehensive Computational Study,” Journal of Orthopaedic Research 40, no. 9 (2022): 2209–2221. 34981558 10.1002/jor.25244

